# Microscopic Insects, Apparently Developed from Inorganic Matter, by Galvanism

**Published:** 1837

**Authors:** 


### Art. IV.—Microscopic Insects, apparently developed from inorganic matter, by Galvanism.

We are indebted to the Journal of Science of our distinguished countryman, Professor Silliman, for a sketch of some very curious experiments, by Mr. Crosse, of England. They are stated by Mr. Scutchbury, in a communication to the Bristol Institution, and were transcribed out of a private letter from the experimenter himself.

The following is an accurate account of the experiments in which insects made their appearance:—

*Experiment first.*—I took a dilute solution of silicate of potash, supersaturated with muriatic acid, and poured it into a quart basin, resting on a piece of mahogany; a Wedgwood funnel was placed in such a manner that a strip of flannel, wetted with the same, and acting as a siphon, conveyed the fluid, drop by drop, through the funnel, upon a piece of somewhat porous Vesuvian red oxide of iron, which was thus kept constantly wetted by the solution, and across the surface of which (by means of two platina wires connected with the opposite poles of a voltaic battery, consisting of nineteen pair of five-inch plates in cells filled with water and 1-500 muriatic acid,) a constant electric current was passed. This was for the purpose of procuring crystals of silex. At the end of fourteen days, I observed two or three very minute specks on the surface of the stone, white, and somewhat elevated. On the eighteenth day, fine filaments projected from each of these specks, or nipples, and the whole figure was increased in size. On the twentysecond day, each of these figures assumed a more definite form, still enlarging. On the twenty-sixth day, each assumed the form of a perfect insect, standing upright on four or five bristles, which forms its tail. On the twenty-eighth day, each insect moved its legs, and in a day or two afterwards detached itself from the stone, and moved at will. It so happened that the apparatus was placed fronting the south, but the window opposite was covered with a blind, as I found these little animals much disturbed when a ray of light fell on them; for out of about fifty which made their appearance at once, at least forty-five took up their habitation on the shaded side of the stone. I ought to have added, that when all the fluid, or nearly so, was drawn out of the basin, it was caught in a glass bottle, placed under a glass funnel, which supported the stone, and was then returned into the basin without moving the stone. The whole was placed on a light frame made for the purpose. These insects have been seen by many of my friends, and appear, when magnified, very much like cheese-mites, but from twice to eight times the size, some with six legs, others with eight. They are covered with long bristles, and those at the tail, when highly magnified, are spiny. After they had been born some time they became amphibious, and I have seen them crawl about on a dry surface.

*Experiment second.*—I took a saturated solution of siliciate of potash, and filled a small glass jar with it, into which I plunged a stout iron wire, connected with the *positive* pole of a battery of twenty pair of cylinders, filled with water alone, and immersed in the same a small coil of silver wire, connected with the *negative* pole of the same battery. After some weeks' action, gelatinous silex surrounded the iron wire, and, after a longer period, the same substance filled up the coil of silver wire at the other pole, but in much less quantity. In the course of time, one of these insects appeared in the silex at the negative pole, and there are at the present time not less than three well-formed precisely similar insects at the negative, and twelve at the positive pole; in all fifteen. Each of them is deeply imbedded in the gelatinous silex, the bristles of its tail alone projecting, and the average of them are from half to three quarters of an inch below the surface of the fluid.

In this last experiment we have neither *acid*, nor *wood*, nor *flannel*, nor *volcanic iron-stone.* I will not say whether they would have been called to life without the electric agency or not. *I offer no opinion, but have merely stated certain facts.*

On these new and unexpected results, Prof. Silliman has the following remark:

We cannot believe that life and organization have been produced by galvanic power; but would sooner suppose that the ova of the insects may have been contained in the materials galvanized, or come into them during the process, and that the galvanic power may have quickened them into life, as electricity and animal warmth operate upon eggs.

We entirely concur in this explanation, as consonant with analogy, and much less incredible, than the hypothesis, of the actual communication to mineral particles, by the galvanic influence, of a tendency to organization and vital movement. The close resemblance which the new animalculæ bear, to those which were previously known, strengthens our opinion. Moreover, we as yet perhaps know but little of the invisible world of animal and vegetable life; many whole races, countless in numbers, and diversified forms and their *modus propagationis*, may remain to be discovered. They or their germs or ova may, from their very minuteness, float perpetually in the atmosphere, or attach themselves to solids or fluids, in any and every situation to which the air can have access. The size of microscopic insects, can furnish no argument for their origin on a principle different from that, on which those are reproduced, which are visible to the naked eye. D.

